# Measuring the compressive modulus of elasticity of pith-filled plant stems

**DOI:** 10.1186/s13007-017-0250-y

**Published:** 2017-11-09

**Authors:** Loay A. Al-Zube, Daniel J. Robertson, Jean N. Edwards, Wenhuan Sun, Douglas D. Cook

**Affiliations:** 1 Division of Engineering, New York University-Abu Dhabi, P.O. Box 129188, Abu Dhabi, United Arab Emirates; 2 Faculty of Engineering, The Hashemite University, P.O. Box 330127, Zarqa, Hashemite Kingdom of Jordan; 3 Department of Mechanical Engineering, University of Idaho, 875 Perimeter Drive, MS 0902, Moscow, Idaho 83844-0902 USA

**Keywords:** Corn stalk tissue, Compression stiffness, Strain measurement

## Abstract

**Background:**

The compressional modulus of elasticity is an important mechanical property for understanding stalk lodging, but this property is rarely available for thin-walled plant stems such as maize and sorghum because excised tissue samples from these plants are highly susceptible to buckling. The purpose of this study was to develop a testing protocol that provides accurate and reliable measurements of the compressive modulus of elasticity of the rind of pith-filled plant stems. The general approach was to relying upon standard methods and practices as much as possible, while developing new techniques as necessary.

**Results:**

Two methods were developed for measuring the compressional modulus of elasticity of pith-filled node–node specimens. Both methods had an average repeatability of ± 4%. The use of natural plant morphology and architecture was used to avoid buckling failure. Both methods relied up on spherical compression platens to accommodate inaccuracies in sample preparation. The effect of sample position within the test fixture was quantified to ensure that sample placement did not introduce systematic errors.

**Conclusions:**

Reliable measurements of the compressive modulus of elasticity of pith-filled plant stems can be performed using the testing protocols presented in this study. Recommendations for future studies were also provided.

## Background

The measurement of mechanical properties of plant stems helps in investigating early and late season stalk lodging [1]. But in spite of the economic significance of plants with thin-walled stems (e.g., maize, sorghum, wheat, etc.), few studies have investigated reliable methods for obtaining their mechanical properties under compressive loading. One of the most important mechanical properties is the modulus of elasticity, which provides a linear relation between stress and strain [2]. This mechanical property is essential for calculating stress states as well as physical deformation of a structure or a plant [3, 4].

The modulus of elasticity can be measured in a number of ways, including bending, tension, compression, vibration, and acoustic excitation tests. Bending tests have been used in a number of studies, including those focused on the mechanical properties of wood [5–8], sunflower stalks [9], sorghum stalks [10], wheat stems [11], and maize stalks [12, 13]. Bending tests are popular because they involve low loads, easily measurable deformation, and require little sample preparation. Bending tests can only be performed on test samples that are long and slender [14], and produce one estimate of the modulus of elasticity for each sample. As a result, this method produces rather poor spatial resolution for the modulus of elasticity. The accuracy of the modulus obtained by bending tests is also adversely affected by the nonlinear form of the bending equations, which tends to amplify measurement uncertainty.

Tensile testing is another common technique for obtaining the modulus of elasticity. This approach has been used to measure the modulus of elasticity of wood [7], excised rind sections of maize stems [15, 16], excised longitudinal sections of switchgrass stems [17], rice stems, and Arabidopsis stems [18]. However, sample preparation is more laborious as compared to bending and specimens must be gripped securely without inducing tissue damage. The gripping aspect of tensile test is often quite challenging.

Compression testing is very common in the wood literature [7, 19], but is not commonly used in the testing of thin-walled plant stems. This is because the plant rind tends to be highly susceptible to buckling deformation. Consequently, information on the compressive modulus of thin-walled plant stems is often not available.

Studies have reported that the tensile and compressive modulus of elasticity values can be different for lumber, wheat straw, and barley straw [6, 20]. This indicates that tensile testing alone may be insufficient for measuring the modulus of elasticity, and that bending tests (which induce both bending and compression) may yield modulus values which are unreliable. Techniques for measuring the compressive modulus of elasticity of plant stems are therefore needed.

For thin-walled plant stems, bending loads are primarily borne by longitudinal stresses in the rind tissue [21, 22], so this study focuses on the longitudinal modulus of elasticity.

The goal of this study was to develop a robust method for obtaining the compressive modulus of elasticity of the rind of pith-filled plant stems, and to study the factors that influence the accuracy and reliability of this method. For the sake of brevity, the abbreviated term “modulus of elasticity” will be used in place of the more precise term “longitudinal compressive modulus of elasticity” in the remainder of this paper.

## Methods

### Stalk samples

Dry maize stalks were used as test specimens in this study. Maize can be highly susceptible to late-season stalk lodging, which occurs due to compression-induced buckling of the rind [13]. Maize stalks were sampled from 2 replicates of four commercially available hybrids of dent corn (maize) seeded at 5 planting densities (119,000, 104,000, 89,000, 74,000, and 59,000 plants ha^−1^) [23]. Stalks were cut just above the ground and just above the ear node immediately before harvest. To prevent fungal growth, stalks were placed in forced-air dryers to reduce stalk moisture to approximately 10–15% moisture by weight, which closely mimics the state of stalks in the field just prior to harvest. To avoid confounding factors, only stalks found to be free of disease and pest damage were included in the study. One hundred (100) samples were selected for compression testing.

### CT scanning

**Fig. 1:**
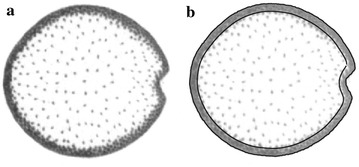
Transverse cross-section of a maize stalk as obtained by X-ray computed tomography. **a** X-ray CT image, **b** X-ray CT image overlaid with lines used to segment the image into rind and pith regions. Segmentation was performed with a custom computer algorithm [23]

X-ray computed tomography was used to quantify cross-sectional areas of the rind and pith regions (Fig. 1). Stalks were scanned using an X5000 scanner (NorthStar Imaging, Rogers, MN, USA). The scanning process produced 2D cross-sectional images of the maize stalks. A customized computer program was used to extract the cross-sectional area of each stalk from the CT data. The scanning and morphology extraction are described in more detail in a previous study [23].

### Sample preparation

**Fig. 2:**
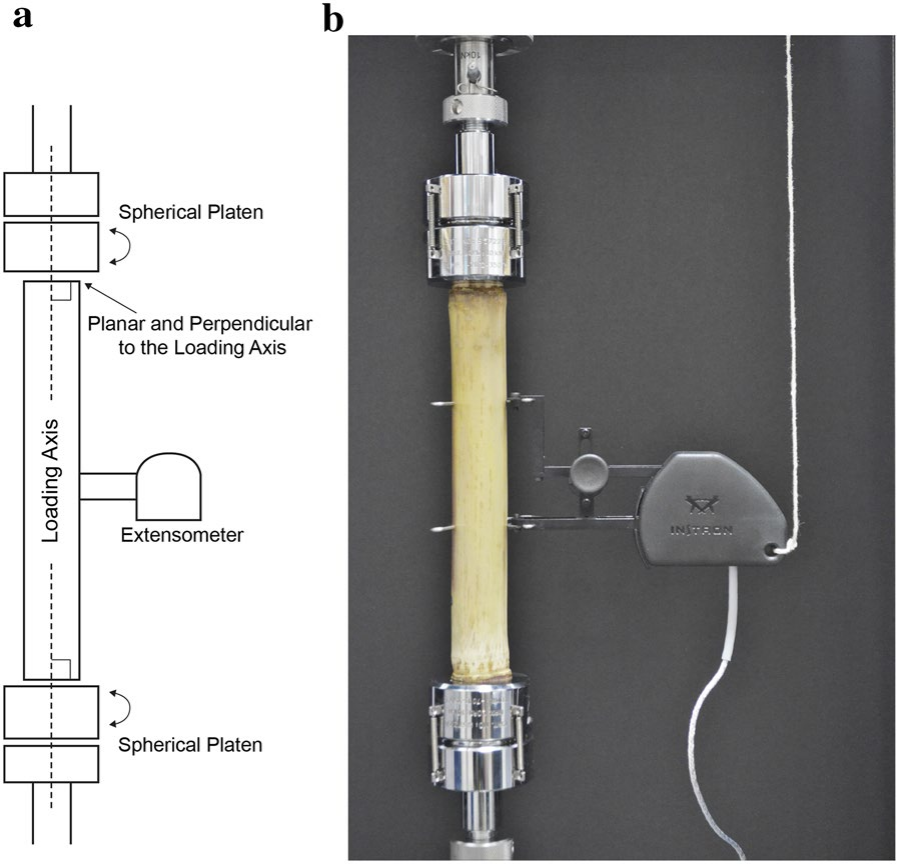
Compressive testing setup: **a** schematic diagram depicting geometric features of an ideal compression test; **b** a photograph of one specimen situated for testing

Technical standards have been developed for compression tests on metals [24], plastics [25], and biomaterials [26]. Each of these standards specifies that sample geometry is critical for accurate assessment of compressional stiffness. These standards require that samples are prepared with end faces that are planar and perpendicular to the loading axis (Fig. 2a). This insures that stresses applied during testing are evenly distributed throughout the specimen.

**Fig. 3:**
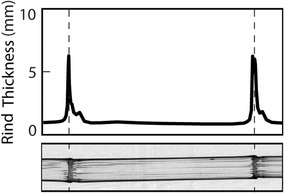
(Top) Maize rind thickness as a function of axial distance. (Bottom) X-ray computed tomography image of the corresponding maize stalk. Dashed lines indicate the locations where the rind is thickest. Samples were prepared by cutting near the dashed lines. A prepared sample is shown in Fig. 2b

In this study, test specimens were cut from stalks with an abrasive saw (Bosch GCO2000, Gerlingen, Germany). The face of the rotating saw blade insured that end faces were planar. Because the rind is thickest just below the node line [13], specimens were cut just below each node, as shown in Fig. 3. This approach utilizes the natural architecture of the stalk to minimize the stresses applied to each end during testing. Specimens prepared in this manner were found to be very durable, thus enabling the performance of multiple tests on individual specimens without induced permanent tissue damage. The prepared specimens contained three distinct tissue regions, each of which differed in anatomy and geometry; internode tissue, elongation zone tissue, and a subapical primary elongation meristem region [27].

Self-aligning compression platens are used in situations where the perpendicularity of sample end-faces is difficult to achieve. As a load is applied, these platens rotate until they are in alignment with the testing surface, thus accommodating any discrepancies in the angle of the end-face. Self-aligning platens (Cat No: S5722A, Instron Corp., Norwood, MA, USA) were therefore used at both ends of the specimens to accommodate any angular inaccuracies in the cutting process. Figure 2 provides a diagram and a photograph of a specimen situated for testing.

### Compression testing equipment

Compression tests were performed using a universal testing machine (Instron 5965, Instron Corp., Norwood, MA, USA). Loads were measured with a 5 kN Instron load cell. Instrumentation control and data acquisition were managed with Instron software (Bluehill 3.0).

Two types of strain were measured for each sample in this study; overall strain (*ε*_*overall*_) and local strain (*ε*_*local*_). Overall strain was based on the total displacement of the universal testing machine, (i.e., the displacement between the two spherical platens) divided by the total initial length of the sample prior to loading. Local strain was measured using an Instron extensometer, which recorded the displacement of two points on the surface of the specimen (see Fig. 2). The extensometer had a reference length of 50 mm (Instron 2630 Series Dynamic Extensometer, Instron Corp., Norwood, MA, USA) (Fig. 2).

### Compression testing procedure

When testing biological tissues, a preload and repeated application of load cycles is commonly used to bring the samples to a repeatable reference state [28]. This procedure is used to reduce measurement variability and is referred to as pre-conditioning [28–31]. The loading process is described below.

An initial load of 200 N was applied to each specimen. Five loading cycles were then applied. In each loading cycle, the load increased from 200 to 700 N and then returned to the 200 N initial state. The first cycle was used as a conditioning cycle. Only measurements from the latter four cycles were employed in the modulus of elasticity calculations. A strain rate of 0.1 mm/s and a sampling frequency of 33 Hz were used in this study. This rate is similar to that used in a previous report (0.0833 mm/s), where corn stalk specimens with a length to diameter ratio of 1:1 were tested [20]. Lower rates have been used in testing wheat/barley straw (0.04 mm/s) [20], lumber (0.005 mm/s) [7] and timber (0.042 mm/s) specimens [32]. Further investigation is needed in the future to elucidate the effect of strain rate on the compressive elastic moduli values of pith-filled plant stems.

### Modulus of elasticity calculations

Compressive modulus is defined as the slope of a uniaxial stress–strain curve. Because the rind is the primary load-bearing tissue of the maize stalk [33], the compressional stress, σ, was obtained by dividing the applied force, *F*, by the cross-sectional area of the rind, *A*_*r*_ (Eq. 1). Cross-sectional areas were measured 5 cm below the node.

1 $$\sigma = \frac{F}{{A_{r} }}$$

This approach neglects the structural contribution of the pith tissue, but allows the estimation of the rind stiffness from a single test. As will be shown in the results section, this assumption introduces relatively minor errors.

For small deformations, strain is obtained by dividing the change in length by the original length:

2 $$\varepsilon = \frac{\Delta L}{{L_{0} }} = \left( {\frac{{L_{f} - L_{0} }}{{L_{0} }}} \right)$$

The slope of the stress–strain curve, or compressive modulus, *E*, was calculated as follows:

3 $$E = \frac{\Delta \sigma }{\Delta \varepsilon } = \frac{{\sigma_{2} - \sigma_{1} }}{{\varepsilon_{2} - \varepsilon_{1} }} = \frac{{\left( {\frac{{F_{2} - F_{1} }}{{A_{r} }}} \right)}}{{\left( {\frac{{L_{2} - L_{1} }}{{L_{0} }}} \right)}} = \left( {\frac{\Delta F}{\Delta L}} \right) \frac{{L_{0} }}{{A_{r} }}$$

In this equation, ΔF and ΔL values in this study corresponded to the changes measured between F_1_ = 200 N and F_2_ = 700 N. Equation 3 was used to compute overall and local compressive modulus of each sample.

**Fig. 4:**
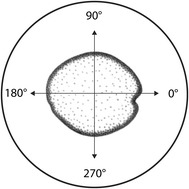
Top view of the self-aligning platen with a maize stalk cross-section and angular directions of strain measurement

The above equations represent a standard approach to measuring the compressive modulus. Although the self-aligning platens accommodated non-perpendicular end-faces, the self-aligning nature of these platens in combination with the complex geometry of the stalk was found to cause circumferential variation in strain distribution within the specimens. To account for potential variations in stress, local strain measurements were obtained from 4 equally spaced positions around the circumference of each specimen, denoted by the angular position of each measurement: *ε*_0_, *ε*_90_, *ε*_180_, and *ε*_270_ (see Fig. 4). Equation 3 was used to calculate corresponding Compressive Modulus values (*E*_0_, *E*_90_, *E*_180_, and *E*_270_).

These individual strain values were combined to obtain a more accurate assessment of the compressive modulus. Because strain is inversely related to the compressive modulus, special attention must be paid to the manner in which averaging is performed [34–37]. Instead of inserting individual strain values into Eq. 3, the strain values were first averaged to obtain a single average strain value (ε_local_) representing the average cross-sectional strain:

4 $$\varepsilon_{local} = \frac{1}{4}\left( {\varepsilon_{0} + \varepsilon_{90} + \varepsilon_{180} + \varepsilon_{270} } \right)$$

This strain can be substituted into Eq. 3 as follows to obtain a final expression for the local compressive modulus, *E*_*local*_:

5 $$E_{local} = \frac{\Delta \sigma }{{\Delta \varepsilon_{local} }} = \frac{{\left( {\frac{{F_{2} - F_{1} }}{{A_{r} }}} \right)}}{{\frac{1}{4}\left\{ {\left( {\frac{{L_{2} - L_{1} }}{{L_{0} }}} \right)_{0} + \left( {\frac{{L_{2} - L_{1} }}{{L_{0} }}} \right)_{90} + \left( {\frac{{L_{2} - L_{1} }}{{L_{0} }}} \right)_{180} + \left( {\frac{{L_{2} - L_{1} }}{{L_{0} }}} \right)_{270} } \right\}}}$$

As before, the subscript indices 1 and 2 refer to the test conditions at loads of 200 and 700 N, respectively.

### Assessing the contribution of pith tissue

After all specimens were tested, the contribution of pith tissue to overall stiffness was assessed by carefully drilling a hole of 5 mm in diameter through the nodal tissue at the end-face of each specimen. A common wood drill bit was used for this purpose. A round wood file was then used to gently abrade the pith tissue until only rind tissue remained. These hollow samples were then re-tested using the techniques described above.

### Sensitivity of the compressive modulus to sample placement

The cross-sectional shape of the maize stalk is somewhat irregular (see Fig. 4). Placement of each specimen on the two self-aligning platens is therefore somewhat subjective. The sensitivity of the compressive modulus measurements to specimen placement was therefore assessed to determine if sample placement affected compressive modulus results.

These tests were performed by first placing a specimen at the apparent center of each self-aligning platen. After measuring the compressive modulus in the typical fashion, the specimen was shifted away from the center and the test was repeated. This process was repeated for shift distances of 2 mm and 4 shift directions (0°, 90°, 180°, and 270°). The compressive modulus was therefore measured at each of the 12 resulting shift locations. These measurements were balanced by 12 tests performed with the specimen in the center position. Testing alternated between centered and shifted positions to avoid potential bias caused by temporal effects.

### Statistical analysis

#### Measurement repeatability

Repeatability of the compression test methodologies in this paper was performed according to standard procedures [38]. A set of 10 specimens were tested repeatedly according to the protocols described above. Each specimen was tested 5 times, and both methods for obtaining compressive modulus were used for each test. The standard deviation was used to quantify the test repeatability for each specimen.

## Results

### Representative stress–strain curves of pith-filled maize samples

**Fig. 5:**
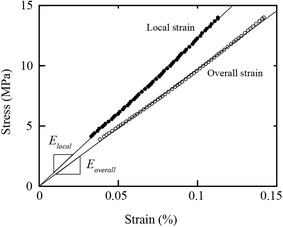
Representative stress–strain curves for local and overall measurements. Slopes of each curve represent the respective compressive modulus values, *E*_*local*_, and *E*_*overall*_

The stress–strain curves for both overall and local compressive modulus values were linear in nature. The loads used in this study typically resulted in strain values less than 0.5%. Representative curves are shown in Fig. 5, which illustrates that the stiffness measured via local strain measurements was generally higher than the overall stiffness. For all tests in this study, the coefficient of determination (*R* ^2^) between stress and strain was above 0.99.

### Sensitivity of the compressive moduli to pith-filled sample placement

**Fig. 6:**
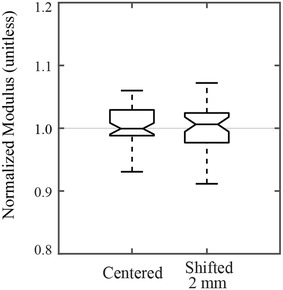
Box plots illustrating the effect of spatial position on specimen placement. All data for a single specimen was normalized by the mean modulus value from the tests conducted at the centered position

Preliminary testing revealed that compressive modulus values were sensitive to sample placement—but only when the sample was shifted more than 2 mm from the center of the platens. The authors’ experience in performing these tests is that a shift of more than 1 mm from the center is easily detectable to the human eye. To assess the influence of spatial position, 10 specimens were tested at the centered position and 2 mm shift positions. Each specimen was tested a total of 8 times: 4 times at the center location, and 4 tests with a 2 mm shift. Each of the 4 “shifted” tests involved shifting the specimen in a different direction, as shown in Fig. 4. The resulting data is shown in Fig. 6, which demonstrates that sample placement within ± 2 mm of the platen center had no significant effect on compressive modulus measurements.

### Repeatability analysis for pith-filled samples

Recall that the test repeatability (i.e., test-to-test variation of a single specimen) was quantified by using the standard deviation for each of 10 samples. Both compression tests methods (using local strain or overall strain) were found to have a mean repeatability of 3.9%. Additional repeatability information is summarized in Table 1. The final column of Table 1 provides an upper bound on test-to-test variation at the 95% confidence level.

**Table 1:** Repeatability statistics obtained from repeated tests on a set of 10 specimens

	Mean repeatability (95% confidence interval)	Repeatability variation (SD) (%)	Upper bound for variation between any two tests (95% confidence) (%)	*n*
*E*_*overall*_	3.9% (± 2.2%)	3.6	11.0	10
*E*_*local*_	3.9% (± 1.7%)	2.7	9.2	10

Five tests were performed on each specimen

### Averaging local strains of pith-filled samples

Self-aligning platens induced slight circumferential variation in strain. This variation was captured by taking strain measurements at 4 circumferential locations for each specimen. To examine the effect of averaging circumferential strains, compressive modulus values were calculated by using 1, 2, 3, and 4 strain values. Equation 3 describes the calculation of compressive modulus for a single strain measurement while Eq. 4 describes the calculation process for four strain measurements. Similar expressions can be obtained for two and three strain measurements.

**Fig. 7:**
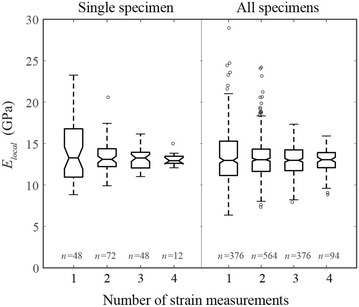
The effect of averaging local strain measurements around the circumference of the test specimens from 1, 2, 3, or 4 sides. Measuring strain from all sides reduces circumferential variation caused by structural asymmetry of the test specimens. Data in this chart is from 94 specimens, with 4 strain measurements per specimen (6 specimens were damaged during testing and therefore were excluded). Sample sizes reflect the number of different combinations for averaging strain measurements (e.g., given 4 strain measurements per specimen, there are 6 possible combinations when using groups of two, 4 combinations when using groups of three, etc.)

The effect of strain averaging is shown in Fig. 7. As expected, variation in the calculated compressive modulus decreased as the number of utilized strain measurement increased. This trend was evident at both the individual and group levels.

### Neglecting the contribution of pith tissue

Several specimens were damaged either during the pith removal process or during subsequent testing, thus reducing the sample size for this portion of the study. The contribution of the pith tissue had a statistically significant effect on the compressive modulus values, which was found to be of approximately the same magnitude as specimen repeatability. The overall mean reduction in stiffness after pith removal was found to be approximately 4%. The variation in this effect was relatively high, which was likely due to the imprecise nature of the pith removal process. At a 95% confidence level, the mean effect of pith tissue on stiffness was found to be less than or equal to 6.3%. We therefore concluded that although the process of neglecting the pith tissue does introduce a consistent error, the magnitude of this error is not substantial. Table 2 provides a summary of the statistics related to pith removal. It is worth noting that the pith prevents failure due to buckling and may therefore significantly contribute to overall stalk strength but not stiffness [39].

**Table 2:** Statistical effects of pith removal

	Mean effect of pith removal (95% confidence interval)	Variation in pith removal (i.e., SD) (%)	*n*
*E*_*overall*_	− 4.4% (± 1.7%)	6.3	54
*E*_*local*_	− 3.8% (± 2.5%)	8.4	47

### Local versus overall compressive moduli of pith-filled samples

We now examine differences between local and overall compressive moduli (*E*_*overall*_ and *E*_*local*_). These values were calculated for all specimens in this study. Recall that *E*_*overall*_ is the stiffness of the entire specimen; whereas *E*_*local*_ is the stiffness obtained near the center of each specimen (see Fig. 2).

**Fig. 8:**
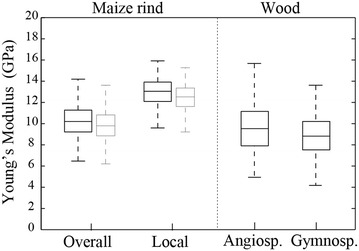
Overall versus local compressive modulus distributions for maize and the two major types of wood. The narrower, gray boxes indicate modulus that have been decreased by the average pith effect of 4%. Wood data from [40, 41]

Figure 8 provides distribution plots for all specimens tested in this study. The mean and standard deviation values for *E*_*overall*_ and *E*_*local*_ were (10.1 ± 1.5 and 12.8 ± 1.5 GPa, respectively). Figure 8 also provides distributions which were shifted downward by 4% to account for the effect of neglecting the pith tissue. Finally, Fig. 8 also provides comparisons to published data on the distribution of the compressive modulus values for dried wood from angiosperms and gymnosperms.

## Discussion

This study involved the compressional testing of dry, non-diseased maize stalk segments consisting of two nodes and the intervening internode (see Fig. 1). Specimens were cut just below the node lines because tough nodal tissues and thicker rind in this region effectively distribute stresses, thus preventing premature tissue failures that can occur during compression testing when test specimens involve only internode tissues.

Certain challenges were encountered in this study. One of these was the difficulty of cutting two parallel end faces on maize stalk specimens, both of which should (according to compression testing standards) be perpendicular to the stalk axis. This challenge was addressed by using two self-aligning compression platens. However, this solution then generated a new challenge: the lack of structural symmetry induced circumferential variation in strain, thus necessitating the measurement of strain at multiple locations. These strains were averaged to obtain the compressive modulus of internodal tissues.

### Accuracy, reliability, and test duration

Two different compressive modulus values were obtained for each specimen in this study: *E*_*overall*_ and *E*_*local*_. The overall compressive modulus value is based on deformation that occurs throughout the entire specimen, including at the end faces, meristematic tissue, and internodal tissues. As such, the overall compressive modulus should be considered as an aggregate stiffness value, with tissue stiffness within the specimen varying above and below this value. The local modulus approach measures tissue strain in a region where tissue is regular and uniform and thus is likely more accurate. Deformation of the testing apparatus was negligible as compared to deformation in test specimens. The repeatability values of both tests were comparable.

The local compressive modulus values were higher than overall modulus values for every specimen in this study. Although spatial variation in stiffness was not the focus of this study, we believe that this is due to a lower tissue stiffness near each node and in the meristematic region [42]. More detailed studies will be necessary to confirm this. The calculation of the compressive modulus values was based on an assumption that the pith tissues have a negligible effect on stalk stiffness. The removal of pith tissue was found to decrease modulus values by an average of 4%. Thus, the values obtained for rind stiffness in this study are (on average) 4% higher than their true values.

As shown in Fig. 7, the reliability of local compressive modulus values improved as the number of circumferential sample points increased. However, unless multiple circumferential samples can be acquired simultaneously, each circumferential sample point increases the testing duration. Excluding sample preparation (which was similar for both test types), local modulus testing required approximately 10 min per specimen.

As shown in Fig. 7, the mean value is relatively insensitive to the number of circumferential strain values. However, a decrease in circumferential measurements also decreases test-to-test repeatability, thus artificially increasing the observed variation in the compressive modulus. The use of fewer circumferential measurements may be suitable in certain situations where the mean value is the primary objective.

If relative differences between plants is of primary concern, absolute accuracy may not be a primary concern. In such a case, the overall modulus may be a better choice. The overall modulus provides a single, average rind stiffness value for the entire specimen with reasonable reliability. Test duration for prepared samples was approximately 2 min per specimen.

### Recommendations for future studies

One of the most important considerations when performing compression tests is the perpendicularity of end faces. This is a particular challenge when dealing with plant stems, which typically do not have straight edges that can be used as a reference. Spherical platens can be used to address this issue, and are recommended for future studies. If for some reason, spherical platens cannot be used, special attention should be paid to the preparation of end faces as well as the resulting load/deformation curves. An alternative approach is to embed each end of the sample in polymethyl methacrylate (PMMA) or some other kind of resin, a technique used in the testing of bone specimens [43, 44].

In the current study, rind thicknesses were obtained from X-ray computed tomography 2D images, but this approach requires special equipment and software. A more accessible technique is to obtain areas of rind and pith areas based on cross-sectional images obtained with a flatbed scanner [45].

## Conclusions

Two methods were developed for measuring the compressive elastic modulus values of the rind of pith-filled plant stems such as maize. The two elastic modulus values were calculated using two different strain measurements. These test methodologies did not require that end faces were strictly parallel, and both methods produced consistent results (mean repeatability of 4%). Both methods utilized the natural shape of the plant stem to avoid stress concentrations and buckling failure which are common challenges when performing compression tests, especially with thin-walled specimens.

Both elastic moduli measurements presented in this study neglected the contribution of the pith tissue. This assumption had a mean effect of overestimating the rind stiffness by 4%, which was deemed to be acceptable for these purposes.

Each of these methods possesses unique advantages and disadvantages. The overall compressive modulus technique provides a single, average value for all rind tissue in the specimen, but can be obtained relatively quickly. In contrast, the measurement of local modulus required multiple strain measurements, thus requiring additional tests, but provided results which are likely more accurate.

The modulus of elasticity values reported in this study are relevant from the stalk-level down to scales of a few centimeters. At scales smaller than this, the cellular architecture of the stalk tissue should be considered. Finally, although these measurements were developed and tested for dry maize specimens, the methods and principles introduced in this study are likely applicable for other types of plant stems, such as sorghum, reed, bamboo, etc.
